# Salt-tolerant and thermostable mechanisms of an endoglucanase from marine *Aspergillus niger*

**DOI:** 10.1186/s40643-022-00533-3

**Published:** 2022-04-21

**Authors:** Li-Nian Cai, Sheng-Nan Xu, Tao Lu, Dong-Qiang Lin, Shan-Jing Yao

**Affiliations:** 1 Key Laboratory of Biomass Chemical Engineering of Ministry of Education, College of Chemical and Biological Engineering, Zhejiang University, Hangzhou, 310027 China; 2 College of Environment, Zhejiang University of Technology, Hangzhou, 310014 China

**Keywords:** Constitutive homologous expression, Endoglucanase, Marine *Aspergillus niger*, Salt tolerance, Thermostability, Salt bridge

## Abstract

The cellulase cocktail of marine *Aspergillus niger* exhibited salt-tolerant and thermostable properties, which is of great potential in industrial application. In order to excavate the single tolerant cellulase components from complex cellulase cocktail, constitutive homologous expression was employed for direct obtainment of the endoglucanase (*An*EGL). Enzymatic property study revealed that *An*EGL exhibited a property of salt tolerance and a strong thermostability in high salinity environment. Significantly, its activity increased to 129% and the half-life at 65 °C increased to 27.7-fold with the presence of 4.5 M NaCl. Molecular dynamics simulation revealed that Na^+^ and Cl^−^ could form salt bridges with charged residues, and then influenced the activity of loops and the stability of substrate binding pocket, which accounted for the salt tolerance and thermostability. Further, site-specific mutagenesis study proved that the residues Asp95 and Asp99 in the pocket were of great concern for the tolerant properties. The salt-tolerant and thermostable *An*EGL was of great value in lignocellulosic utilization and the conjectural mechanisms were of referential significance for other tolerant enzymes.

**Graphical Abstract:**

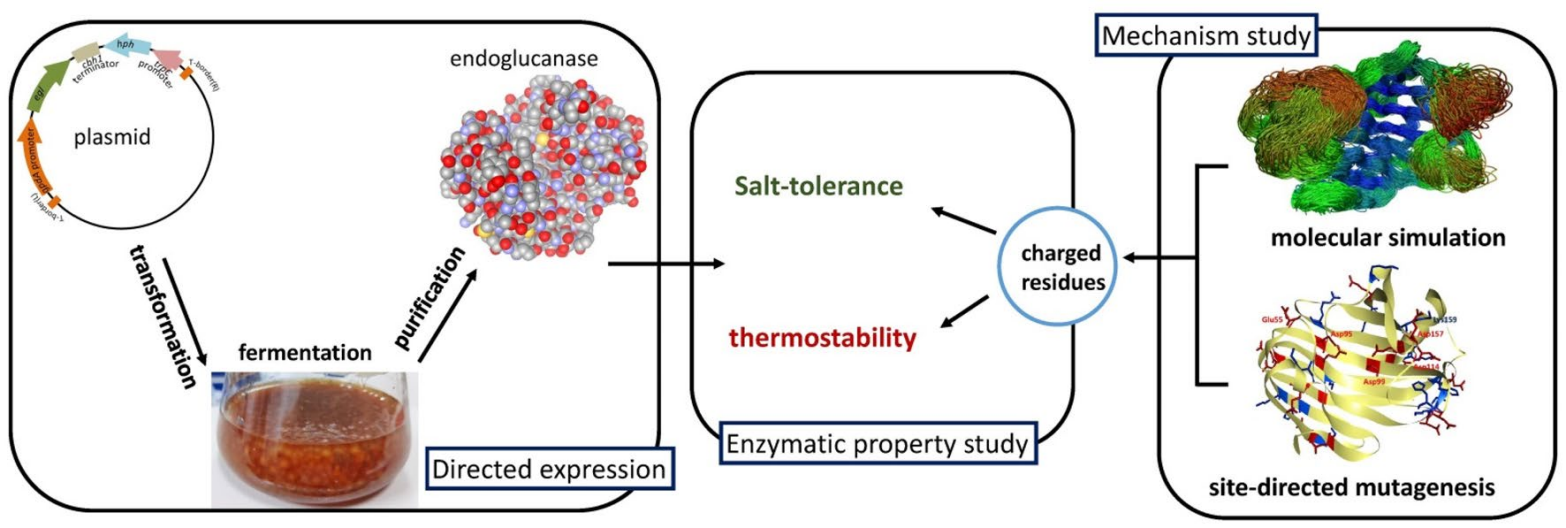

**Supplementary Information:**

The online version contains supplementary material available at 10.1186/s40643-022-00533-3.

## Introduction

Cellulase is an enzyme cocktail that catalyzes the hydrolysis of cellulose to glucose (Payne et al. 2015). Cellulase can be divided into cellobiohydrolase (CBH), endoglucanase (EGL), β-glucosidase (BGL), and lytic polysaccharide monooxygenase (LPMO) (Payne et al. 2015). Enzymatic hydrolysis of cellulose relies on the synergistic action of various cellulases, *i.e.*, CBHs degrade cellulose from both ends of cellulose chain to form cello-oligosaccharides, EGLs and LPMOs act, respectively, on the non-crystalline and crystalline regions of cellulose and degrade the long cellulose chain into short ones, and BGLs degrade the cello-oligosaccharides into glucose (Chylenski et al. 2019; Payne et al. 2015).

Utilization of lignocellulosic resources is one of the potential ways to alleviate or solve energy crisis, in which pretreatment of lignocellulosic biomass and enzymatic hydrolysis of cellulose are two key steps (Hendriks and Zeeman 2009; Soni et al. 2018). Generally, pretreatment may also put some pressure on the enzymatic hydrolysis step (Kumar et al. 2009). Acid/alkali pretreatment makes cellulose substrate rich in salt and ionic liquid pretreatment introduces considerable ions in the substrate. Without desalination, the subsequent enzymatic hydrolysis step is bound to be performed in high salinity environment, which is obviously unfavorable for enzymatic hydrolysis efficiency. Introduction of salt-tolerant cellulases can eliminate or alleviate these adverse effects (DasSarma and DasSarma 2015; Wahlström and Suurnäkki 2015). The salt-tolerant cellulases refer to the ones that can maintain activity even in high salinity condition. Recently, different kinds of salt-tolerant cellulases, including CBHs from glycoside hydrolase family 5 (GH5) and GH7 (Kern et al. 2013; Zhang et al. 2011), BGLs from GH1 and GH3 (Cai et al. 2019; Mai et al. 2013), and EGLs from GH1, GH5, GH8, GH44 and GH45 (An et al. 2015; Deep et al. 2016; Gao et al. 2010; Huang et al. 2010; Mai et al. 2014), have been reported. Among them, the EGLs from GH5 were reported the most. Most of the salt-tolerant cellulases were found from halophiles or in high salinity environment (An et al. 2015; Cai et al. 2019; Huang et al. 2010; Kern et al. 2013). In our previous studies, the cellulases from marine *Aspergillus niger* have been proved to be more salt tolerant than those from terrestrial *A. niger* (Wang et al. 2016; Xue et al. 2012; Xue et al. 2017a, b, c; Xue et al. 2017a, b, c; Xue et al. 2017a, b, c). Fortunately, a salt-tolerant BGL from marine *A. niger* has been successfully characterized (Cai et al. 2019). Some studies have provided speculation on the mechanisms of salt tolerance (Elcock and McCammon 1998; Kern et al. 2013). The generally accepted mechanism is that a large excess of acidic residues distributed in protein surface contribute to bind large amounts of water molecules and metal ions to maintain activity in high salinity. In addition, other factors, such as ordered secondary structure, and salt bridge, also have positive effects on salt tolerance (Madern et al. 2000).

During the enzymatic hydrolysis process, temperature has an important influence on hydrolysis efficiency (Unsworth et al. 2007). As the temperature increases, on one hand, the viscosity of the reaction system decreases and the mass transfer effect is enhanced. On the other hand, the intensification of Brownian motion increases the contact frequency between enzyme and substrate. Therefore, a higher reaction temperature is preferred during enzymatic hydrolysis of cellulose and the thermostability of cellulase is of crucial significance. Thermostable cellulases are widely available from many organisms, including fungi and bacteria (Patel et al. 2019; Shuddhodana and Bisaria 2018). At present, large numbers of thermostable cellulases have been discovered, which are almost exclusively derived from thermophiles (Patel et al. 2019; Shuddhodana and Bisaria 2018). Study on mechanisms of thermostability is of great significance for directed evolution to obtain more thermostable enzymes. Some breakthroughs have been made in study on the mechanisms (Han et al. 2019; Vieille and Zeikus 2001). The thermostability is related with many factors, including amino acid composition, disulfide bond, hydrophobic interaction, aromatic interaction, hydrogen bond, and salt bridge.

For studying on mechanisms of thermostability and salt tolerance, Fourier transform infrared spectroscopy (Fabian et al. 1993), circular dichroism spectrum (Perez-Iratxeta and Andrade-Navarro 2008), and nuclear magnetic resonance spectroscopy (Zhang et al. 2012) can be used to measure the changes in protein secondary structure, and Michaelis constant (*K*_m_) can reflect the substrate binding capacity. However, the deeper mechanisms involving interaction between residues or atoms still cannot be explained clearly. The tertiary structure or the crystal structure of protein is prerequisite but not sufficient for study on the mechanisms. The crystal structure is always obtained under a certain static condition, while the demonstration of thermostability and salt tolerance needs a condition with higher temperature and higher salinity. Molecular dynamics (MD) simulation provides an effective way for dynamic studying the mechanisms of salt tolerance and thermostability (Kadowaki et al. 2018; Kern et al. 2013). By simulating the changes at different salinities or temperatures, it is possible to elucidate the tolerant mechanisms. The changes include secondary structure, salt bridge, solvent accessible surface, hydrogen bond, residue activity, etc.

In this study, we prepared an EGL (*An*EGL) of marine *A. niger* directly from the fermentation broth by homologous constitutive expression. And then *An*EGL with high purity was obtained by simple separation and purification to determine its properties of salt tolerance and thermostability. As an important part, we analyzed the corresponding molecular mechanisms of salt tolerance and thermostability by combining MD simulation with site-directed mutagenesis study.

## Material and methods

### Main strains, plasmids, media, and primers

*A. niger* ZJUBE-1 was stored at the China Center for Type Culture Collection (Conservation No. CCTCC M2010132) and used for homologous expression of *An*EGL. *Agrobacterium tumefaciens* AGL-1 was used for transformation of marine *A. niger*. *Escherichia coli* DH5α and Rosetta (DE3) (Tsingke, Beijing, China) were used for subcloning and heterologous expression of *An*EGL and its mutants. Glucose peptide yeast (GPY) medium (40 g/L glucose, 20 g/L peptone, 5 g/L yeast extract, 4 g/L KH_2_PO_4_) was used for propagation of marine *A. niger* and constitutive expression of *An*EGL. PDA medium (20 g/L potato powder, 20 g/L glucose, 20 g/L agar) was used for the generation and preservation of marine *A. niger*. The induced medium (IM) and minimal medium (MM) used for *A. tumefaciens*-mediated transformation (AMT) of marine *A. niger* were accordant with the protocol (Michielse et al. 2005). The plasmid pCAMBIA-hph-bgl1 was constructed in our previous study (Cai et al. 2019) and the primers used are shown in Additional file 2: Table S1.

### Cloning of endoglucanase genes

About 10^7^ spores of *A. niger* ZJUBE-1 were inoculated in 100 mL GPY medium in 250 mL shake flask and cultivated with shaking at 180 rpm at 30 °C for 3 d. Then the genomic DNA was extracted using DNAiso (Takara, Beijing, China) according to its protocol. The total RNA was extracted with the fungal total RNA isolation kit (Sangon, Shanghai, China) according to its protocol, and then the cDNA was obtained by reverse transcription using oligo(dT)_16_ primer. Subsequently, the EGL genes (*egl1* and *egl2*) were amplified from genomic DNA and cDNA using primers, egl-F and egl-R. Finally, these two EGL genes were inserted into pUCm-T vector (Sangon, Shanghai, China) and the resulting plasmids pUCm-egla/eglb were checked by DNA sequencing using primers, M13F and M13R.

### Construction of Mini-Ti vector

The skeleton of Mini-Ti vector was clone from pCAMBIA-hph-bgl1 using primers, vector-F and vector-R. The target gene *egla* was clone from pUCm-egla using primers, egla-F and egla-R. Then the target Mini-Ti vector pCAMBIA-hph-egla was constructed by ligating these two fragments using pEASY-Uni seamless cloning and assembly kit (TransGen, Beijing, China; this kit was used in the following ligation steps). The DNA fragment PgpdA-egla-Tcbh1-hph-PtrpC was checked by DNA sequencing using primers, sequence-F and sequence-R.

### Transformation of marine *A. niger*

*A. tumefaciens* AGL-1 was transformed with pCAMBIA-hph-egla using freeze–thaw method (Wise et al. 2006). Standard procedures of AMT were used as described in the protocol (Michielse et al. 2005). Briefly, the fresh *A. tumefaciens* recombinant was inoculated in IM broth, cultivated until OD_600_ reached 0.6–0.8. About 100 μL induced *A. tumefaciens* recombinant and 10^6^ spores of marine *A. niger* were mixed and spread on cellophane of IM agar, cultivated for 2 d at 23 °C. Then the cellophane as well as the strains was transferred onto MM agar with 200 μg/mL hygromycin B and 200 μg/mL cefotaxime sodium. Additional MM agar with equal concentration of antibiotics was poured onto the cellophane to enhance screening effect. This interlayer medium was cultivated at 30 °C until recombinants grew out. Positive recombinants were verified by PCR identification using primers, PgpdA-F and PgpdA-R. Single conidiospore isolation was performed for acquisition of homozygote as described in our previous study (Cai et al. 2019). The resulting recombinants were stored at 4 °C.

### Enzyme assay

EGL activity was assayed using sodium carboxymethyl cellulose (CMC-Na) as substrate. The enzymatic reaction mixtures (500 µl) containing 50 µl enzyme solution and 450 µl CMC-Na solution (1% (w/v) CMC-Na, 0.1 M sodium citrate/citric acid, pH 4.0) were incubated for 5 min at 50 °C. The amount of reducing sugar released was determined by 3,5-dinitrosalicylic acid (DNS) assay (Wood and Bhat 1988). One unit of EGL activity was defined as the amount of enzyme required for release 1 µmol glucose equivalent per minute. The values in following text were determined by this method unless otherwise stated.

### Protein expression

For constitutive expression of *An*EGL, 10^7^ spores of recombinant were inoculated in 250 mL GPY medium in 1000 mL shake flask, cultivated with shaking at 200 rpm at 30 °C for 6 d. After fermentation, the supernatant was obtained by filtration with two layers of gauze and concentrated about fivefold by ultrafiltration with molecular weight cut-off 10 kDa. Then the expression was analyzed by sodium dodecyl sulfate–polyacrylamide gel electrophoresis (SDS-PAGE).

### Purification

After constitutive expression, the mycelia were removed using two layers of gauze. Then the supernatant was concentrated and the saline ions as well as the impurities with small molecule were replaced by water using ultrafiltration (molecular weight cut-off 10 kDa). The pH of ultrafiltrate was adjusted to 7.0 with 0.1 M NaOH and then loaded on DEAE Sepharose column pre-equilibrated with 20 mM sodium phosphate buffer pH 7.0. The fractions were eluted with the gradient of 0–0.5 M NaCl at a flow rate of 1 mL/min. The purity of *An*EGL in each eluent was determined by SDS-PAGE. Then the purified *An*EGL was concentrated and the buffer was replaced by 20 mM sodium acetate buffer pH 4.0 by ultrafiltration (molecular weight cut-off 10 kDa). The resulting enzyme solution was stored at 4 °C.

### Enzymatic properties

The purified *An*EGL was used for the study on enzymatic properties. In terms of pH, the enzymatic reaction mixtures (500 μL), including 50 μL properly diluted enzyme solution and 450 μL CMC-Na solution (1% (w/v) CMC-Na, 0.1 M sodium citrate/citric acid, pH 2.5, 3.0, 3.5, 4.0, 4.5, 5.0, 5.5, 6.0, and 6.5), were incubated in water bath at 50 °C for 5 min. In terms of temperature, the enzymatic reaction mixtures (500 μL), including 50 μL properly diluted enzyme solution and 450 μL CMC-Na solution (1% (w/v) CMC-Na, 0.1 M sodium citrate/citric acid, pH 4.0), were incubated in water bath at different temperatures (20, 30, 40, 50, 60, 70, and 80 °C) for 5 min. In terms of metal ions (5 mM and 10 mM), the enzymatic reaction mixtures (500 μL), including 50 μL properly diluted enzyme solution, 5 μL or 10 μL metal ions solution (NaCl, Na_2_SO_4_, NaNO_3_, LiCl, AgNO_3_, MgCl_2_, CaCl_2_, NiCl_2_, CuSO_4_, ZnSO_4_, CrCl_2_, HgSO_4_, FeCl_3_, 0.5 M), and 445 μL or 440 μL CMC-Na solution (1% (w/v) CMC-Na, 0.1 M sodium citrate/citric acid, pH 4.0), were incubated in water bath at 50 °C for 5 min. In terms of salt concentration, the enzymatic reaction mixtures (500 μL), including 50 μL properly diluted enzyme solution and 450 μL CMC-Na solution (1% (w/v) CMC-Na, 0, 1.0, 2.0, 3.0, 4.0, 5.0 M NaCl, 0.1 M sodium citrate/citric acid, pH 4.0), were incubated in water bath at 50 °C for 5 min. In terms of thermostability, 50 μL properly diluted enzyme solution was added into 450 μL CMC-Na solution (1% (w/v) CMC-Na, 0.1 M sodium citrate/citric acid, pH 4.0) after incubated at 50, 60, 65, and 70 °C for 5, 10, 15, 20, 30, 40, 50, 60, 90, and 120 min. Then the enzymatic reaction mixtures were incubated in water bath at 50 °C for 5 min. In terms of the influence of salt concentration on thermostability, enzyme solution was diluted properly with 0.1 M sodium citrate/citric acid buffer pH 4.0 with different NaCl concentrations (0, 1.0, 2.0, 3.0, 4.0, 5.0 M), incubated at 65 °C for 4, 8, 12, 16, 20, 30, 60 min. Subsequently, 50 μL of the diluted enzyme solution was added into 450 μL CMC-Na solution (1% (w/v) CMC-Na, 0.1 M sodium citrate/citric acid, pH 4.0). Then the enzymatic reaction mixtures were incubated in water bath at 50 °C for 5 min. The highest EGL activity was defined as 100%.

### MD simulation

The amino acid sequence of *An*EGL was translated from the sequence of *egl2*, and then its signal peptide was predicted by SignalP (http://www.cbs.dtu.dk/services/SignalP-4.0/). The alignment of amino acid sequences with other solved EGLs was accomplished with DNAMAN 8. The homology modeling of *An*EGL was accomplished with Discovery Studio 3.0 using the crystal structure of *A. niger* β-1,4-endoglucanase (EglA, PDB ID: 1KS4) as template (Khademi et al. 2002). The conserved substrate binding sites and catalytic residues were determined according to the solved structure of EglA. The MD simulation was performed with GROMACS at two temperatures (300 and 350 K) and three salt concentrations (0, 2, and 4 M NaCl). After simulation, the root mean square deviation (RMSD), root mean square fluctuation (RMSF), and salt bridges were calculated by GROMACS. The visualization of trajectories was accomplished by VMD.

### Obtainment of mutant *An*EGLs

In order to simplify protein expression and enzyme assay, the mutant *An*EGLs as well as original *An*EGL were expressed in *E. coli* Rosetta (DE3). Firstly, the plasmid pET-eglb used for expression of original *An*EGL was constructed. Briefly, the *eglb* encoding mature peptide was amplified from pUCm-eglb using primers, eglb-F and eglb-R. The linearized pET28a was obtained by PCR using primers, pET-F and pET-R. The plasmid pET-eglb was constructed by ligating these two fragments. Secondly, the plasmids pET-mutant1/2/3/4/5/6/7 were constructed. In detail, the entire plasmid pET-eglb was amplified using seven pairs of primers (mutant1-F and mutant1-R, mutant2-F and mutant2-R, mutant3-F and mutant3-R, mutant4-F and mutant4-R, mutant5-F and mutant5-R, mutant6-F and mutant6-R, mutant7-F and mutant7-R), respectively. The amplified products were transformed into *E. coli* Rosetta(DE3) after digested with restriction endonuclease *Dpn*I (Takara, Beijing, China). Thirdly, the plasmid pET-mutant8 was constructed. Briefly, four fragments of *eglb* were amplified from pET-eglb using primers, mutant8-1-F and mutant8-1-R, mutant8-2-F and mutant8-2-R, mutant8-3-F and mutant8-3-R, and mutant8-4-F and mutant8-4-R, respectively. The linearized pET28a was obtained by PCR using primers, pET8-F and pET8-R. The plasmid pET-mutant8 was constructed by ligating these five fragments. The eight recombinant plasmids were transformed into *E. coli* Rosetta(DE3). The positive mutant EGL genes were identified by DNA sequencing using primers, T7F and T7R.

As for protein expression, the recombinants were inoculated into 5 mL LB broth, cultivated with shaking at 200 rpm at 37 °C overnight. The culture was inoculated into 50 mL LB broth, cultivated at 37 °C 200 rpm until OD_600_ reached 0.4–0.6. Isopropyl-β-D-thiogalactopyranoside (IPTG) was added into the culture to 0.1 mM of final concentration, cultivated with shaking at 200 rpm at 16 °C for 16 h. After expression, the crude enzyme solutions were obtained by cell disruption with ultrasonic wave. The influences of salt concentration and temperature on activity were determined as mentioned in “Material and methods” section of enzymatic properties.

## Results

### Cloning of endoglucanase genes

The EGL genes (*egla* and *eglb*) were cloned into pUCm-T vector and sequenced. Considering that *egla* and *eglb* were cloned from genomic DNA and cDNA, respectively, it could simply locate the introns and exons by comparing the sequences of *egla* and *eglb*. The sequence of *egla* containing splicing sites of intron–exon has been submitted to GenBank (Accession No. MK587440). Considering that splicing sites of intron–exon and peptide can be well recognized in marine *A. niger*, the *egla* gene was directly inserted into the Mini-Ti vector for homologous expression.

### Constitutive expression and purification

**Fig. 1:**
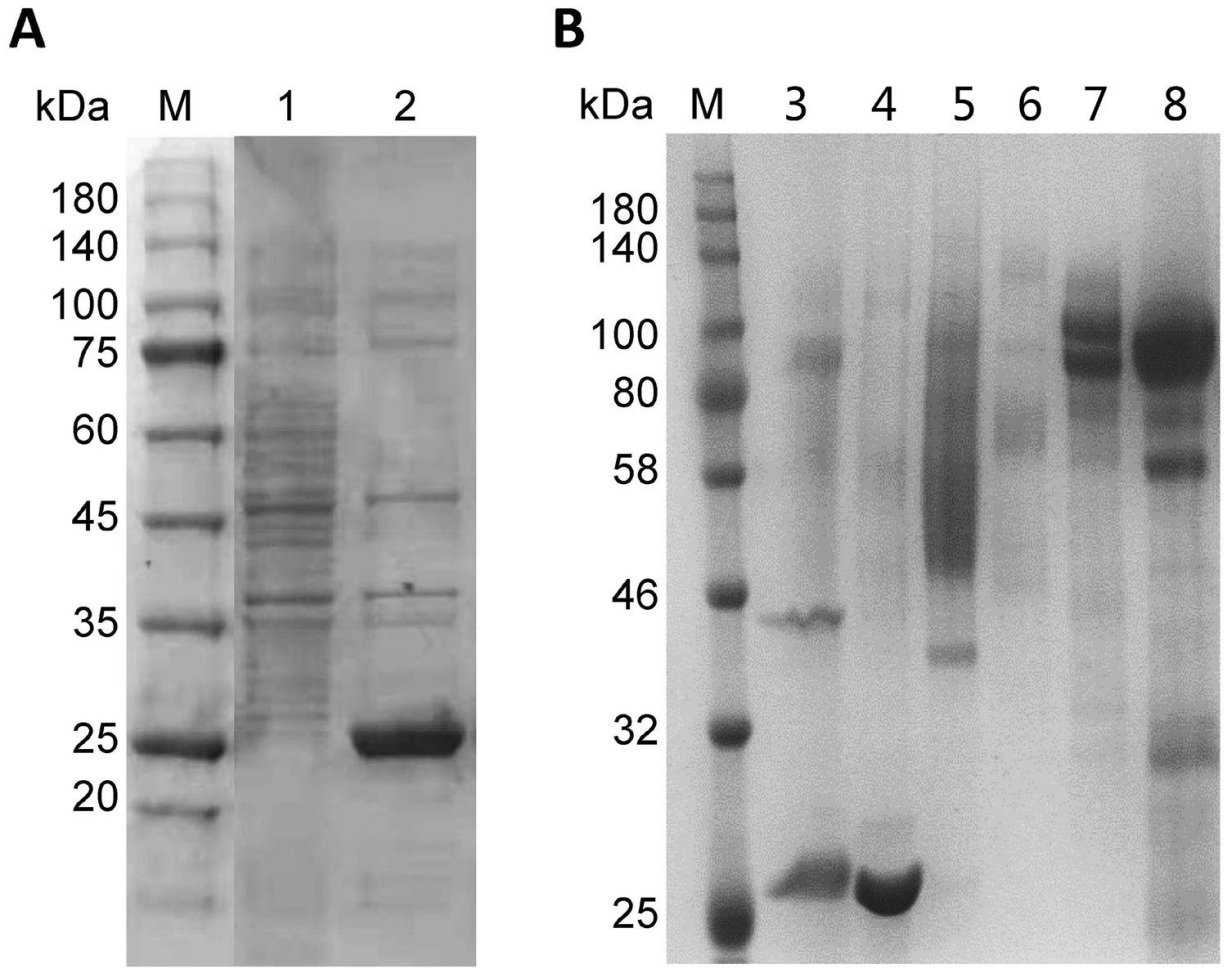
Constitutive expression and purification. **A** Constitutive expression in GPY medium. Lane M: protein marker; lane 1: concentrated supernatant of original strain; lane 2: concentrated supernatant of recombinant. **B** Purification of *An*EGL by anion exchange chromatography. Lane M: protein marker; lane 3: ultrafiltrate; lane 4: wash with 20 mM pH 7.0 sodium phosphate buffer; lanes 5 to 8: elution with 0.1, 0.2, 0.3, 0.5 M NaCl

The constitutive expression of *An*EGL in GPY medium was analyzed by SDS-PAGE. Obvious bands were observed in the supernatant of recombinant at molecular weight of about 26 kDa (Fig. 1A). Considering that EGL activity was detected in the fermentation broth when cultivated the original strain in GPY medium, it is necessary to purify the crude *An*EGL before studying on its enzymatic properties. For the few contaminants in fermentation broth, the purification of *An*EGL was accomplished by one step of anion exchange chromatography. The *An*EGL was eluted with 20 mM sodium phosphate buffer pH 7.0 and the contaminants were eluted with 0.1–0.5 M NaCl (Fig. 1B). Although no pre-elution was employed, the purity of *An*EGL was still very high. In addition, gradient elution with NaCl was performed on the supernatant of the original strain, and it was found that the impurity with EGL activity was concentrated in the eluent of 0.2–0.3 M NaCl (Additional file 2: Fig. S1). Therefore, the purified *An*EGL could be used for the subsequent study.

### Enzymatic properties

**Fig. 2:**
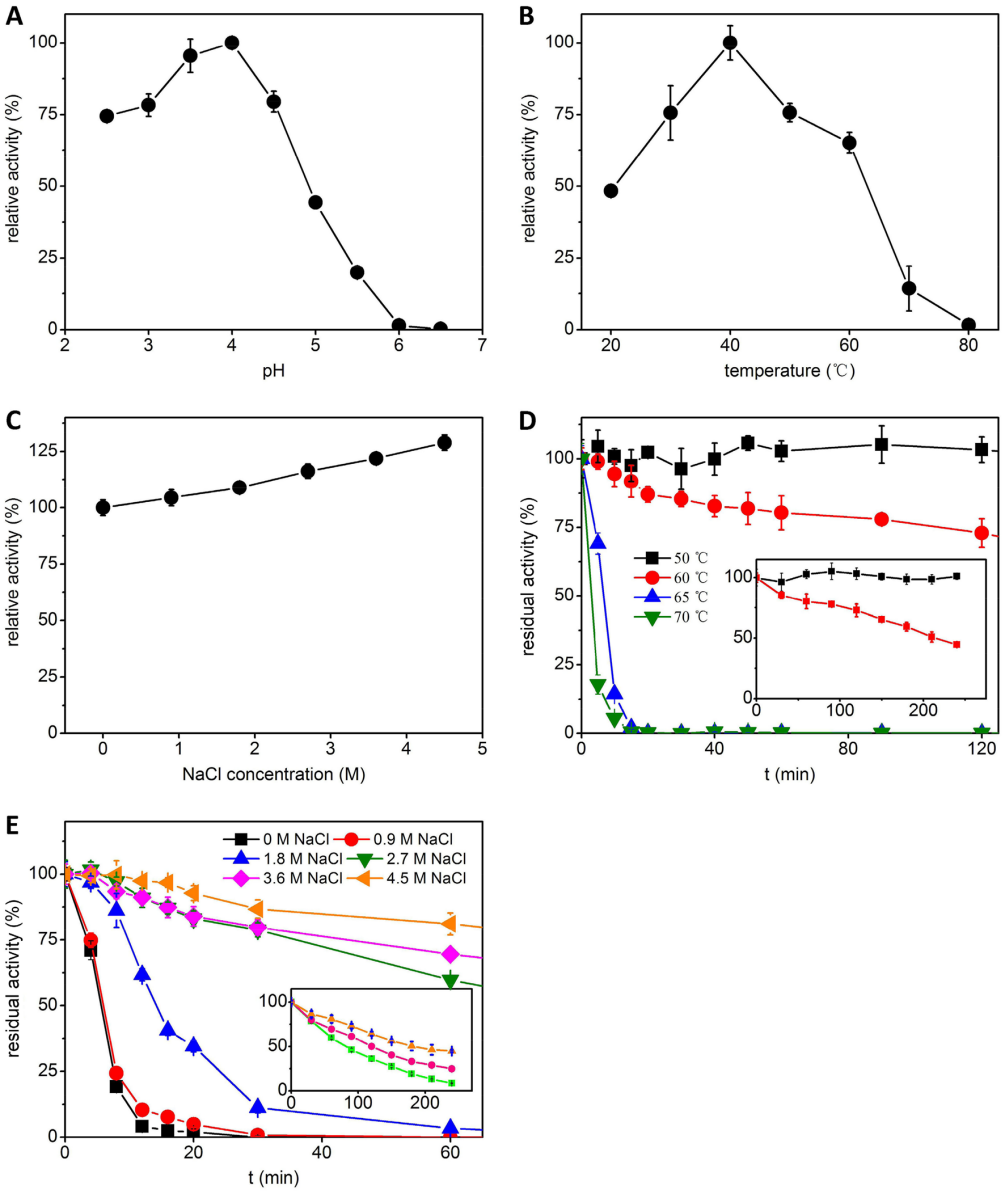
Enzymatic properties of *An*EGL. **A** Influence of pH on the activity at 50 °C in the absence of NaCl. **B** Influence of temperature on the activity at pH 4.0 in the absence of NaCl. **C** Influence of salt concentration on the activity at 50 °C pH 4.0. **D** Thermostability at pH 4.0 in the absence of NaCl. The horizontal axis (incubated time) was expanded in the small graph. **E** Influence of salt concentration on the thermostability at 65 °C pH 4.0. The horizontal axis (incubated time) was expanded in the small graph

The influence of pH on the activity of *An*EGL is shown in Fig. 2A. The purified *An*EGL showed that maximal activity at pH 3.0–4.0 and retained 75% of activity at pH 2.5. However, the activity decreased sharply when pH was over 4.5 and was hardly detected when pH was up to 6.0. The influence of temperature is shown in Fig. 2B. It showed that the optimal activity was 40 °C. The influence of salt concentration is shown in Fig. 2C. The activity increased as the NaCl concentration increased and reached the maximum (increased to 129%) in the presence of 4.5 M NaCl, suggesting that *An*EGL had a property of salt tolerance. The thermostability of *An*EGL is shown in Fig. 2D. The activity kept constant when *An*EGL was incubated at 50 °C for 4 h. The half-lives of *An*EGL were about 210, 6.5 and 2.5 min at 60, 65, and 70 °C, respectively. The activity was completely abolished after incubation at 65 °C for 20 min or at 70 °C for 15 min. It seemed that the thermostability was not very different from that of mesophilic EGLs. The influence of salt concentration on the thermostability of *An*EGL is shown in Fig. 2E. As the NaCl concentration increased, *An*EGL retained higher activity after incubation. The half-lives of *An*EGL were about 6.5, 14, 80, 120, and 180 min at 65 °C in the presence of 0.9, 1.8, 2.7, 3.6, and 4.5 M NaCl, respectively. The thermostability of *An*EGL was enhanced with the increase of NaCl concentration. Especially, when NaCl concentration was higher than 3.6 M, the thermostability of *An*EGL was comparable to the EGLs from thermophiles (Patel et al. 2019). The influences of metal ions on the activity of *An*EGL are shown in Table 1. It was found that the activity of *An*EGL remained near 100% when 5 mM/10 mM NaCl, Na_2_SO_4_, and NaNO_3_ were added into the reaction mixtures, which revealed that the influences of different anions on activity of *An*EGL were inconspicuous. Among various metal ions (5 mM) tested for their influences on the activity of *An*EGL (Table 1), a better enhancement (119%) of activity was observed with 5 mM Mg^2+^, but the activity was strongly inhibited by Ag^+^, Cu^2+^, and Hg^2+^. When the concentration was up to 10 mM, *An*EGL became sensitive to most metal ions except with Na^+^, Mg^2+^, and Fe^3+^. The activity was almost undetectable in the presence of 10 mM Ag^+^, Cu^2+^, and Hg^2+^. Heavy metal ions are highly reductive and can react with the thiol groups of cysteine residues of proteins, which is the most common mechanism of protein inactivation (Barbara 2008). In addition, Khademi et al. (2002) studied the irreversible inhibition mechanism of Pd^2+^ on EGL activity and found that Pd^2+^ forms a coordinate covalent bond with Met and Glu at the active site of the enzyme. The inhibition mechanism of Ag^+^, Cu^2+^, and Hg^2+^ on the activity of *An*EGL might be the same as the mechanisms mentioned above.

**Table 1:** The influence of metal ions on the activity of *An*EGL

Metal ions	Relative activity (%)		Metal ions	Relative activity (%)	
	5 mM	10 mM		5 mM	10 mM
CK^a^	100	100	Mg^2+^ (MgCl_2_)	119	101
NaNO_3_	102	101	Ni^2+^ (NiCl_2_)	98	58
Na_2_SO_4_	101	99	Cu^2+^ (CuSO_4_)	76	8
NaCl	100	101	Zn^2+^ (ZnSO_4_)	105	85
Li^+^ (LiCl)	105	82	Hg^2+^ (HgSO_4_)	29	4
Ag^+^ (AgNO_3_)	54	5	Fe^3+^ (FeCl_3_)	108	97
Ca^2+^ (CaCl_2_)	107	67	Cr^3+^ (CrCl_3_)	98	83

^a^The control check, which was not added with metal ions

### Sequence alignment and homology modeling

**Fig. 3:**
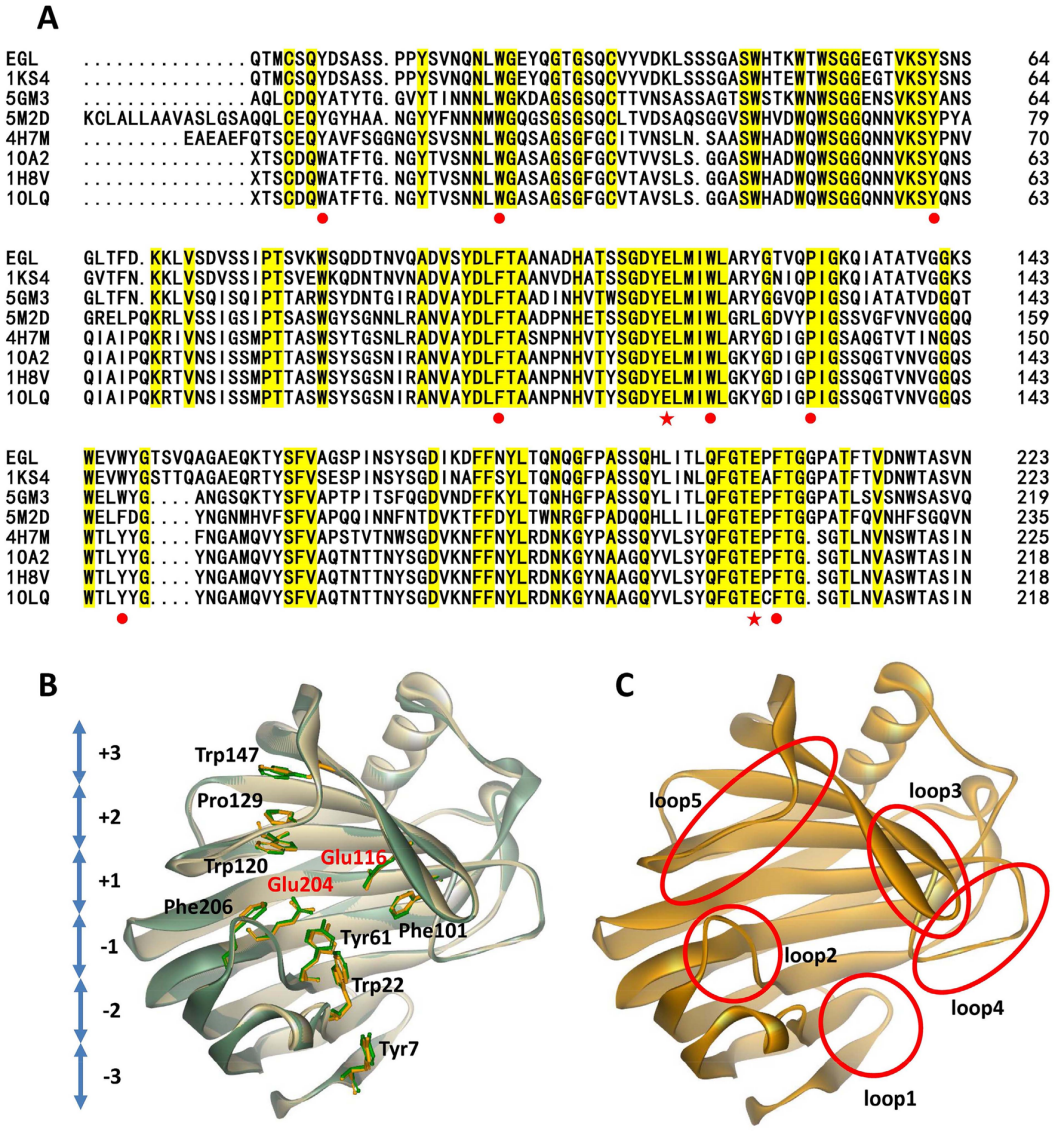
Analysis on sequence and structure of *An*EGL. **A** Amino acid sequence alignment of EGLs. 1KS4, 5GM3, 5M2D, 4H7M, 1OA2, 1H8V, and 1OLQ are the PDB IDs of EGLs, 1KS4: EGL from *A. niger*, 5GM3: EGL from *Aspergillus aculeatus*, 5M2D: EGL from *Acremonium chrysogenum*, 4H7M: EGL from *Trichoderma harzianum*, 1OA2, 1H8V, and 1OLQ: EGLs from *Trichoderma reesei*. The residues with 100% homology level are highlighted with yellow. The substrate binding residues and catalytic residues of *An*EGL are marked with red solid circles and pentagrams, respectively. **B** Modeling structure and key residues of *An*EGL. The structures of EglA and *An*EGL are displayed with light green and light orange, respectively. Key residues of EglA and *An*EGL are displayed with green and orange, respectively. **C** Distribution of key loops involved in substrate binding

Sequence alignment and homology modeling of *An*EGL are shown in Fig. 3A and B and the modeled structure is provided in Additional file 1: AnEGL.pdb. *An*EGL showed higher homology to EglA (89.7% identity), which belonged to GH12 (Khademi et al. 2002). The overall fold of *An*EGL strongly resembled that of EglA, which had a “jelly-roll” fold with two antiparallel sheets (Khademi et al. 2002). As shown in Fig. 3B, the substrate binding sites and their spatial position from subsite − 3 to + 3 (Tyr7, Trp22, Tyr61, Phe101, Phe206, Trp120, Pro129, and Trp147) were consistent with those of EglA (Khademi et al. 2002). Besides, the catalytic residues, Glu116 and Glu204, were highly conserved throughout the EGLs from GH12 (Fig. 3A). Here, we noticed that most of the substrate binding residues located in loops. For the sake of analysis, the loops were numbered from loop 1 to loop 5 (Fig. 3C). Although the homology between *An*EGL and the reported EglA was very high, the study on EglA did not involve the properties of salt tolerance and thermostability.

### MD simulation

**Fig. 4:**
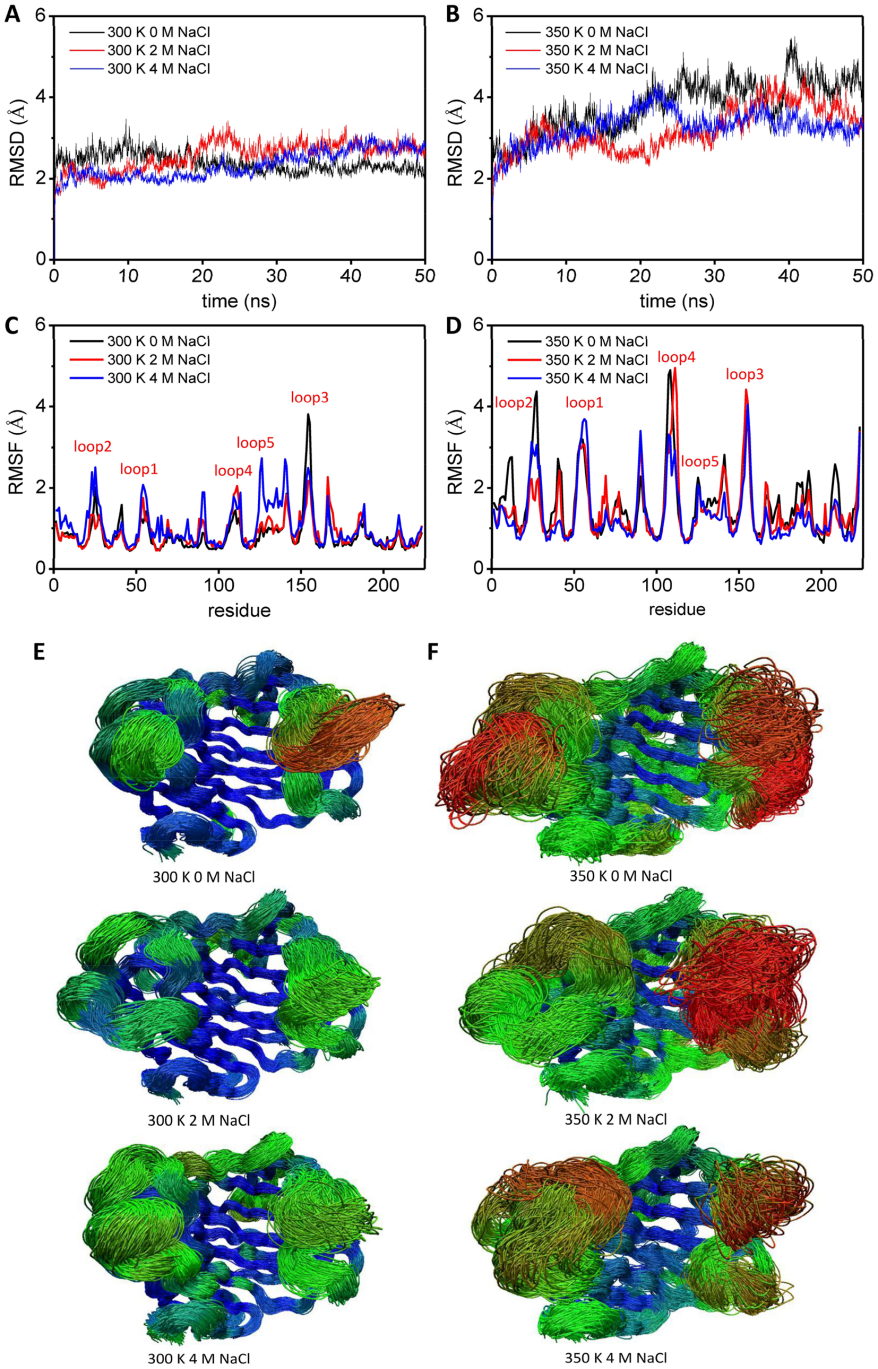
RMSD and RMSF analyses at different temperatures and NaCl concentrations. **A** RMSD of *An*EGL at 300 K within 50 ns MD simulation. **B** RMSD of *An*EGL at 350 K within 50 ns MD simulation. **C** RMSF of C-α at 300 K within 20 to 50 ns MD simulation. **D** RMSD of C-α at 350 K within 20 to 50 ns MD simulation. **E** Activity analysis on *An*EGL at 300 K within 20 to 50 ns MD simulation. **F** Activity analysis on *An*EGL at 350 K within 20 to 50 ns MD simulation. The proteins are colored according to the RMSF of atom within 20 to 50 ns MD simulation. The activity from strong to weak is shown as colored gradient from red to blue

**Fig. 5:**
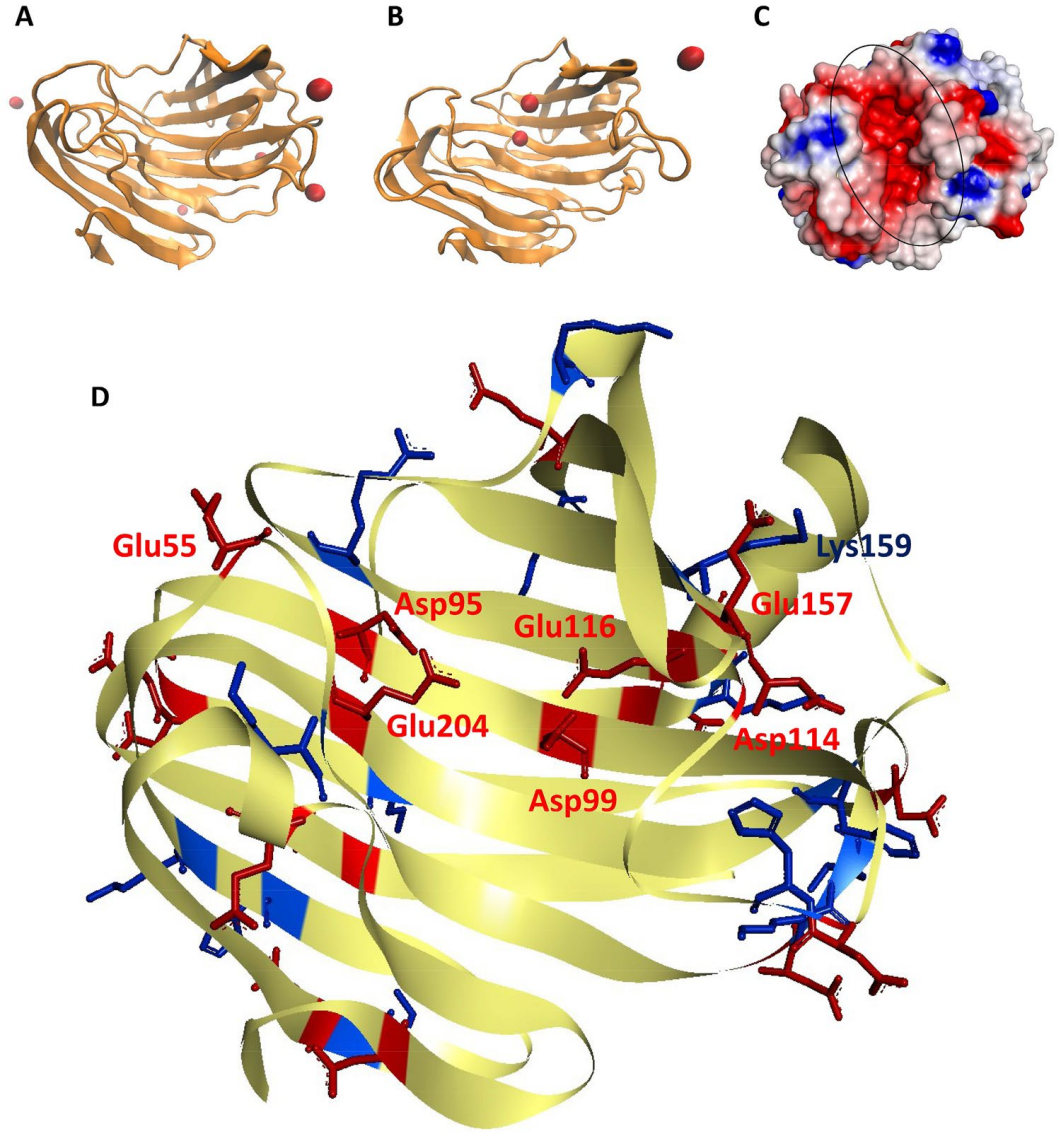
The function of NaCl on salt tolerance and thermostability. **A** The spatial position of Na^+^ before MD simulation at 300 K in the absence of NaCl. **B** The spatial position of Na^+^ after 5 ns MD simulation at 300 K in the absence of NaCl. The red sphere represents Na^+^. **C** Surface electrostatic potential of *An*EGL. The region circled with the ellipse is the substrate binding pocket. Electrostatic potential between − 5 kT/e and 5 kT/e is shown as colored gradient from red to white to blue. **D** Charged residues of *An*EGL. The electropositive and electronegative residues are colored with blue and red, respectively

MD simulation was performed at different temperatures and salt concentrations in order to study the salt-tolerant and thermostable mechanisms of *An*EGL. The RMSD results are shown in Fig. 4A (300 K) and Fig. 4B (350 K). At 300 K, the RMSD achieved a balance quickly after about 5 ns MD simulation at different NaCl concentrations. At 350 K, its structure kept deviating from the initial position in the absence of NaCl, while this deviation tendency was relieved with the increase of NaCl concentration. The structure tended to be stable after 30 ns and 10 ns in the presence of 2 M and 4 M NaCl, respectively. The RMSF results are shown in Fig. 4C (300 K) and Fig. 4D (350 K). At 300 K, with the increase of NaCl concentration, the regions of residue 22–29, 52–57, 87–92, 107–113, and 123–144 became active, while the region of residue 152–157 became inactive. At 350 K, things are different. The regions of residue 22–29, 107–113, and 207–213 were most active in the absence of NaCl. The positions of near the 90th residue (residue 87–92) and the 210th residue (residue 207–213) are shown in Additional file 2: Fig. S2. In comparison from Fig. 4C and D, the floating in these two regions was less significant, and the floating of the region near the 210th residue probably related with the adjacent carboxyl end of *An*EGL. In addition, these two regions were relatively far away from the substrate binding pocket, whose effect on catalysis was limited. Hence, these two regions were not considered in the salt-tolerant and thermostable mechanisms. In order to better demonstrate the activity of different regions under different conditions, the trajectories of *An*EGL during 20 to 50 ns were overlapped and colored by RMSF of carbon atoms (Fig. 4E and F). The regions of residue 22–29, 52–57, 107–113, 123–144, and 152–157 corresponded to loop 2, 1, 4, 5, and 3, respectively. At 300 K, except for loop 3, the other loops showed a more active trend with the increase of NaCl concentration, of which loop 5 was the most obvious one. At 350 K, the activity of loops 1 to 4 decreased with the increase of NaCl concentration. In addition, it was found that in the absence of NaCl, the substrate binding pocket tended to be exposed and the structure deviated significantly from the initial one at 350 K (Fig. 5F).

### Salt tolerance and thermostability of mutant *An*EGLs

According to the MD results, eight mutant *An*EGLs (mutant 1: D95N; mutant 2: D99N; mutant 3: E55Q; mutant 4: D114N; mutant 5: E157Q; mutant 6: K159T; mutant 7: D95N, D99N; mutant 8: E55Q, D114N, E157Q, K159T) were designed. The expression of *An*EGL and eight mutant *An*EGLs in *E. coli* is shown in Additional file 2: Fig. S3. The apparent molecular weights of *An*EGL and eight mutant *An*EGLs were accordance with the *An*EGL homologously expressed, which suggested that no glycosylation occurred in heterologous expression. Considering that no EGL activity was detected in original *E. coli*, the crude enzyme solutions prepared with ultrasonication were directly used for subsequent determination of salt tolerance and thermostability.

**Fig. 6:**
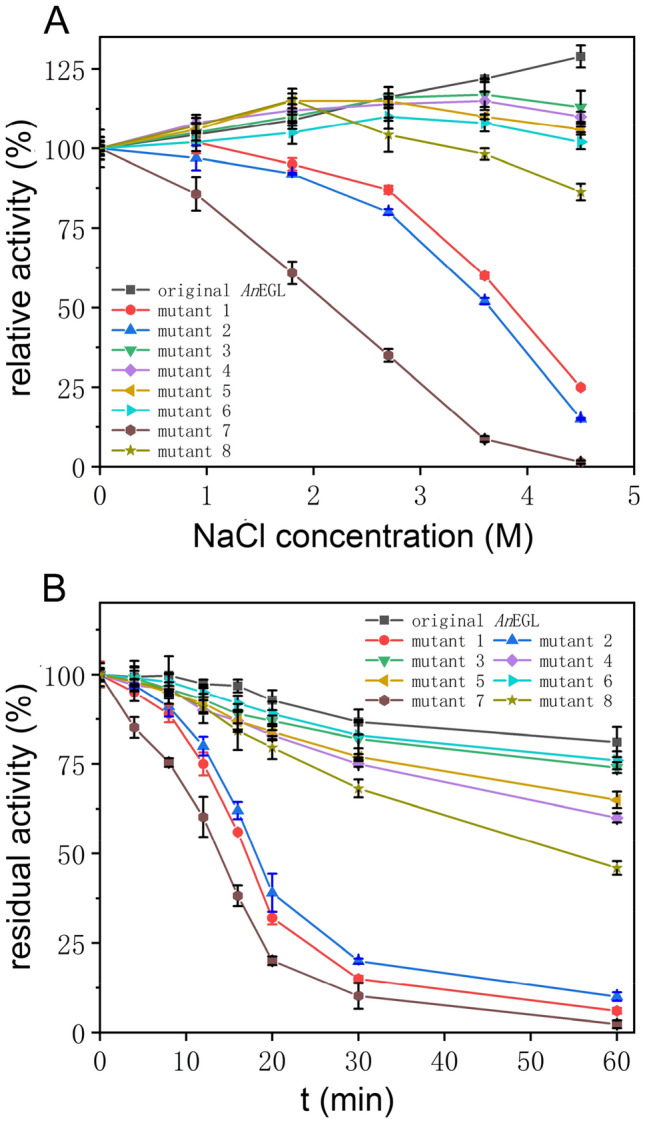
The salt tolerance and thermostability of *An*EGL and its mutants. **A** Influence of NaCl concentration on activity at 50 °C pH 4.0. **B** The thermostability at 65 °C pH 4.0 with the presence of 4.5 M NaCl. The mutation sites of mutant 1–8: mutant 1 (D95N), mutant 2 (D99N), mutant 3 (E55Q), mutant 4 (D114N), mutant 5 (E157Q), mutant 6 (K159T), mutant 7 (D95N, D99N), mutant 8 (E55Q, D114N, E157Q, K159T)

The influence of salt concentration on EGL activity is shown in Fig. 6A. It was found that the activity of mutant 7 significantly decreased with the increase of NaCl concentration. At the condition of 4.5 M NaCl, its activity could be scarcely detected. Mutants 1 and 2 were slightly recalcitrant to high salinity. Their activity could retain more than 80% when NaCl concentration was below 2.7 M, while decreased sharply when NaCl concentration was above 2.7 M. Comparatively, the activity of mutants 3–6 and 8 was less affected by salt concentration. The influence of salt concentration on thermostability of mutants is shown in Fig. 6B. Compared with the activity of *An*EGL, when incubated at 65 °C in the presence of 4.5 M NaCl, the activity of mutants 1, 2, and 7 sharply decreased. After incubated at 65 °C for 60 min, mutant 7 was almost deactivated. In comparison, the activity of mutants 3–6 and 8 decreased more slowly. After incubated at 65 °C for 60 min, the activity of mutant 8 retained 45.9%.

## Discussion

Cellulase is an enzyme cocktail and produced in the presence of inducer. As a result, there are always various components of cellulases in fermentation broth. Direct separation to the cellulase cocktail is hardly competent to obtain a single cellulase with high purity. Here, we adopted constitutive expression to prevent the expression of inducible cellulases, which was named as “directed expression” (Cai et al. 2019). The expression cassettes contained the *gpdA* promoter, the target genes, and the *cbh1* terminator, which realized constitutive expression in marine *A. niger*. It is convenient to obtain a single enzyme from a complex inducible enzyme system by using this constitutive expression system**.**

Acid treatment is widely used in the pretreatment of lignocellulosic biomass (Kumar et al. 2009). However, the treated biomass will be acidic without neutralization. *An*EGL exhibited an acidophilic property, which conferred it with the potential to hydrolyze cellulose under acidic condition, and then reduce the cost of neutralization. In addition, *An*EGL exhibited the property of salt tolerance and strong thermostability in high salinity environment, which would greatly expand its application, especially in cellulose hydrolysis with high salinity and high temperature. Moreover, *An*EGL was the first reported GH12 EGL with salt-tolerant property. Some salt-tolerant EGLs are summarized in Table 2, most of which belonged to GH5.

**Table 2:** Salt-tolerant and salt-philic EGLs

Cellulase name	Source	GH family	pH optimum	Temperature optimum (°C)	Salt concentration optimum	Relative activity (%)	References
EG1	*Stachybotrys microspora*	NM^a^	7.0	50	5 M NaCl	152	Ben Hmad et al. (2017)
endoglucanase	*Haloarcula* sp. G10	NM	9.0	60	3 M NaCl	NC^b^	Li and Yu (2013)
egl01	*Bacillus licheniformis*	5	5	50	2–3 M NaCl/KCl	> 120	Hua et al. (2015)
EG-PY2	*Paenibacillus* sp. Y2	NM	4.5	30	0.5 M NaCl	211.5	Lee et al. (2017)
Cel5A	*Vibrio* sp. G21	5	6.5–7.5	50	0.5 M NaCl	160	Gao et al. (2010)
En5H	*Alkalilimnicola* sp. NM-DCM1	5	8.8	55	2.5 M NaCl	500	Mesbah and Wiegel (2017)
MgCel44	mangrove soil metagenomic library	44	6.0	45	0.5 M NaCl	160	Mai et al. (2014)
AgCMCase	*Aspergillus glaucus* CCHA	5	5.0	55	1.0–4.0 M NaCl	230	Li et al. (2018)
*An*EGL	*A. niger* ZJUBE-1	12	3.5–4.0	40	4.5 M NaCl	129	This study

^a^Not mentioned

^b^The relative activity cannot be calculated because there was no activity in the absence of NaCl

RMSD and RMSF results revealed that high salinity environment could activate some loops at a mild temperature (300 K) and stabilize the structure at high temperature (350 K). Many substrate binding sites were located on the loops, and the loops with proper activity were of important guarantee for substrate binding (Khademi et al. 2002). The relative active loops in high NaCl concentration might be the reason why the activity of *An*EGL increased slightly with the increase of NaCl concentration. Overactive loops might result in some irreversible changes and permanent loss of enzyme activity (Yu et al. 2017). At 350 K, with the increase of NaCl concentration, some loops as well as the substrate binding pocket became more stable. Therefore, it could be concluded that high NaCl concentration played important roles in stabilizing the structure of *An*EGL. How did NaCl make the loops stable and active, and how did NaCl make the pocket tend to be closed?

In fact, before performing the MD simulation at 300 K without NaCl, 8 additional sodium ions were added into the system to balance the negative charge. Two of these traces of Na^+^ (approximately 50 mM) entered the substrate binding pocket after 5 ns simulation (Fig. 5A and B) and kept fixed within certain bounds. Similar results were obtained under other simulation conditions. Analysis of the surface electrostatic potential of *An*EGL revealed that the substrate binding pocket was negatively charged (Fig. 5C), which explained the reason why traces of Na^+^ could enter the pocket in such a short time. Subsequently, the charged amino acid residues of *An*EGL were analyzed (Fig. 5D) and it was found that many electropositive residues located in the pocket. Among them, Asp95, Asp99, Glu116, and Glu204 had a catching effect on Na^+^, which could also be reflected by the change of salt bridges between residues and Na^+^ (Additional file 2: Fig. S4). Similar phenomenon that the formation of R-COO^−^···Na^+^ ···^−^OOC-R salt bridges was crucial for enhanced thermostability in high salinity environment has been reported (Liang et al. 2011).

The instability of the loops mainly comes from two aspects, one is the interaction of the internal residues and the other is the interaction between the residues of loops and the molecules in solution (Vieille and Zeikus 2001). At mild temperature, the interaction between Na^+^ and residues of loops was weaker than that of the internal residues. Thus, at 300 K, protein structure as well as loop activity did not change much in different NaCl concentrations. The decreased activity of loop 3 may be due to the elimination of the interaction between loop 3 and loop 4 by Na^+^ and Cl^−^, which made the force acting on loop 3 tends to be simple and ultimately reduced its activity. There was only one Lys residue (Lys132) in loop 5 and as the Cl^−^ concentration increased, the frequency of interaction between Cl^−^ and loop 5 increased, thereby increasing its activity. At 350 K, the activity of the whole protein increased. At this time, the frequency of interaction between Na^+^ and Cl^−^ and loops increased accordingly. As the concentration of NaCl increased, the force between the loops was weakened. Similar to that at 300 K, the activity of loop 3 was greatly reduced. In detail, salt bridges could be formed between Lys159 in loop 3 and Glu157 in loop 3 or between Lys159 in loop 3 and Asp114 in loop 4. Due to the limitation of steric hindrance and the interference of Na^+^ and Cl^−^, the salt bridge between Lys159 and Asp114 became unstable with the increase of NaCl concentration (Additional file 2: Table S2), which weakened the interaction between loop 3 and loop 4.

Because the substrate binding pocket was rich in electronegative residues, the pocket exhibits strong negative charge. In the pocket, in addition to Asp95, Asp99, Glu116, and Glu204, there were other electronegative residues, such as Glu55 in loop 1, Glu157 in loop 3, and Asp114 in loop 4. If there was not enough Na^+^ to balance the negative electricity, the electronegative residues would produce a strong repulsive force to expand the pocket. At 300 K, due to the lower activity of Na^+^ and residues, two Na^+^ were basically located in the pocket during the simulation, and thereby, the expansion of the pocket was not obvious. When the temperature was up to 350 K, the activity of Na^+^ increased, and the captured Na^+^ might escape from the pocket. When the Na^+^ concentration was low, the negative charge of the pocket was not well balanced, so the pocket tended to expand. With the increase of Na^+^ concentration, despite the escape of Na^+^, the Na^+^ outside the pocket would enter the pocket due to the differential concentration. Thus, the pocket did not expand to a large extent in high NaCl concentration. Further, site-directed mutagenesis was employed to verify the mechanisms conjectured by MD simulation. It could be concluded that (1) the charged residues both in substrate binding pocket and loops contribute the salt tolerance and thermostability of *An*EGL and (2) the charged residues in substrate binding pocket were more requisite for the strong salt tolerance and thermostability than those in loops.

## Conclusions

In this study, the strategy of directed expression was employed for efficient obtainment of the target enzyme *An*EGL. *An*EGL exhibited a salt-tolerant activity and a strong thermostability in high salinity environment, which was potential and competitive in industrial application. MD simulation revealed that the salt bridges formed between charged residues and Na^+^ and Cl^−^influenced the activity of loops and the stability of pocket, and then conferred on *An*EGL the salt tolerance and strong thermostability in high salinity environment, which were verified by site-directed mutagenesis. These conjectural mechanisms were of reference value for the study on salt tolerance and thermostability.

### Supplementary Information

**Additional file 1: **AnEGL.

**Additional file 2:** **Table S1.** Primers used in this study. **Table S2.** Changes of salt bridges within 20 to 50 ns simulation. **Table S3.** Some thermostable endoglucanases from various microorganisms. **Figure S1.** NaCl gradient elution to the supernatant of original strain. **Figure S2.** The positions of the regions near the 90th and 210th residues. **Figure S3.** The expression of *An*EGL and its mutants in *E. coli*. **Figure S4.** Changes of salt bridges within 20–50 ns simulation.

## Data Availability

The data generated and/or analyzed during this study are available from the corresponding author on reasonable request.

## Abbreviations

BGL: β-Glucosidase
EGL: Endo-1,4-β-glucanase
CBH: Cellobiohydrolase or exo-1,4-β-glucanase
LPMO: Lytic polysaccharide monooxygenase
AMT: *Agrobacterium tumefaciens*-mediated transformation
GH: Glycoside hydrolase
GPY: Glucose peptide yeast
SDS-PAGE: Sodium dodecyl sulfate–polyacrylamide gel electrophoresis
MD: Molecular dynamics
CMC-Na: Sodium carboxymethyl cellulose
*K*_m_: Michaelis constant
DNS: 3,5-Dinitrosalicylic acid
RMSD: Root mean square deviation
RMSF: Root mean square fluctuation
IPTG: Isopropyl-β-D-thiogalactopyranoside
