# Supplementary material for: Salt-tolerant and thermostable mechanisms of an endoglucanase from marine Aspergillus niger
Source: Bioresour Bioprocess. 2022 Apr 21;9(1):44. doi: 10.1186/s40643-022-00533-3 (PMC10991132; doi:10.1186/s40643-022-00533-3)
Supplement: Supplementary file 2 — Additional file 2: Table S1. Primers used in this study. Table S2. Changes of salt bridges within 20 to 50 ns simulation. Table S3. Some thermostable endoglucanases from various microorganisms. Figure S1. NaCl gradient elution to the supernatant of original strain. Figure S2. The positions of the regions near the 90th and 210th residues. Figure S3. The expression of AnEGL and its mutants in E. coli. Figure S4. Changes of salt bridges within 20–50 ns simulation. [file 40643_2022_533_MOESM2_ESM.docx]

**Supplementary Material**

Journal: *Bioresources and Bioprocessing*

**Halo-tolerant and thermostable mechanisms of an endoglucanase from marine *Aspergillus niger***

*Li-Nian Cai^1^, Sheng-Nan Xu^1^, Tao Lu^2^, Dong-Qiang Lin^2^, Shan-Jing Yao^1*^*

^1^ Key Laboratory of Biomass Chemical Engineering of Ministry of Education, College of Chemical and Biological Engineering, Zhejiang University, Hangzhou 310027, China

^2^ College of Environment, Zhejiang University of Technology, Hangzhou 310014, China

^*^ Corresponding author:

Prof. Shan-Jing Yao

E-mail: yaosj@zju.edu.cn

Tel/Fax: +86-571-87951982

**Table S1** Primers used in this study

| Primers | Nucleotide sequence from 5’ to 3’ |
| --- | --- |
| egl-F | ATGAAGCTCGCTGTGACACT |
| egl-R | TTAGTTGACACTAGCGGTCC |
| oligo(dT)_16_ | TTTTTTTTTTTTTTTT |
| M13F | TGTAAAACGACGGCCAGT |
| M13R | CAGGAAACAGCTATGACC |
| egla-F | GCAGACATCACAATGAAGCTCGCTGTGACACT |
| egla-R | TTTCGCCACGGAGCTTAGTTGACACTAGCGGTCC |
| vector-F | AGCTCCGTGGCGAAAGCCTGACGCA |
| vector-R | CATTGTGATGTCTGCTCAAGCGGGGT |
| sequence-F | CTGCCTGTATCGAGTGGTGA |
| sequence-R | GCACATACAAATGGACGAAC |
| eglb-F | ATGCAGACGATGTGCTCTCA |
| eglb-R | TTAGTTGACACTAGCGGTCC |
| PgpdA-F | GCTTGTATCTCTACACACAG |
| PgpdA-R | TGTGATGTCTGCTCAAGCGG |
| pET-F | GCTAGTGTCAACTAAGATCCGGCTGCTAACAAAGC |
| pET-R | GCACATCGTCTGCATGGTATATCTCCTTCTTAAAGTTAAAC |
| T7F | TAATACGACTCACTATAGGG |
| T7R | GCTAGTTATTGCTCAGCGG |
| mutant1-F | ACCAATGTCCAAGCCAATGTCTCATATGA |
| mutant1-R | TGGCTTGGACATTGGTGTCGTCCTGG |
| mutant2-F | GCCGATGTCTCATATAATCTGTTCACC |
| mutant2-R | TATATGAGACATCGGCTTGGACATTG |
| mutant3-F | CCTGGAGTGGTGGCCAGGGAACAGT |
| mutant3-R | GGCCACCACTCCAGGTCCACTTGGTA |
| mutant4-F | GCCACTTCCAGCGGTAACTATGAGCTT |
| mutant4-R | TACCGCTGGAAGTGGCATGATCCGCA |
| mutant5-F | GTCCAGGCCGGTGCGCAGCAGAAGAC |
| mutant5-R | GCGCACCGGCCTGGACGCTGGTACCA |
| mutant6-F | CGGTGCGGAGCAGACGACATATAGC |
| mutant6-R | GTCTGCTCCGCACCGGCCTGGACGC |
| mutant7-F | TGTCCAAGCCAATGTCTCATATAATCTGTTCAC |
| mutant7-R | TATATGAGACATTGGCTTGGACATTGGTGTCGT |
| pET8-F | ACTGGCCGTCGTTTTACAAC |
| pET8-R | CGTAATCATGGTCATAGCTG |
| mutant8-1-F | ATGACCATGATTACGATGAAGCTCGCTGTGACACT |
| mutant8-1-R | TTTCACTGTTCCCTGGCCACCACTCCAGGTCCACT |
| mutant8-2-F | CAGGGAACAGTGAAAAGTTAC |
| mutant8-2-R | TACCGCTGGAAGTGGCATGAT |
| mutant8-3-F | CCACTTCCAGCGGTAACTATGAGCTTATGATTTGG |
| mutant8-3-R | GTCTGCTGCGCACCGGCCTGGACGCTGG |
| mutant8-4-F | CGGTGCGCAGCAGACGACATATAGCTTCGTGGCAG |
| mutant8-4-R | AAAACGACGGCCAGTTTAGTTGACACTAGCGGTCC |

**Table S2** Changes of salt bridges within 20 to 50 ns simulation

| time (ns) | 350 K 0 M NaCl | | 350 K 2 M NaCl | | 350 K 4 M NaCl | |
| --- | --- | --- | --- | --- | --- | --- |
|  | 114-159^a^ | 157-159 | 114-159 | 157-159 | 114-159 | 157-159 |
| 20 | 6.90^b^ | 7.72 | 5.70 | 6.50 | 4.64 | 2.42 |
| 21 | 4.30 | 4.92 | 6.45 | 6.34 | 7.11 | 3.29 |
| 22 | 4.72 | 5.21 | 6.88 | 6.31 | 6.71 | 5.11 |
| 23 | 6.09 | 6.28 | 8.60 | 5.87 | 10.21 | 4.49 |
| 24 | 6.57 | 8.99 | 7.56 | 9.77 | 12.25 | 4.24 |
| 25 | 4.62 | 4.50 | 6.34 | 6.61 | 8.70 | 5.68 |
| 26 | 6.14 | 4.84 | 6.88 | 8.05 | 10.10 | 5.38 |
| 27 | 3.41 | 6.79 | 7.24 | 6.70 | 8.75 | 3.04 |
| 28 | 4.52 | 5.55 | 5.66 | 5.82 | 10.09 | 4.15 |
| 29 | 4.48 | 3.45 | 6.05 | 7.72 | 11.12 | 2.65 |
| 30 | 3.94 | 3.18 | 8.53 | 7.45 | 9.53 | 7.13 |
| 31 | 4.62 | 2.77 | 5.86 | 7.87 | 9.99 | 6.91 |
| 32 | 5.38 | 4.66 | 9.63 | 6.64 | 9.61 | 3.70 |
| 33 | 7.92 | 6.71 | 9.48 | 7.76 | 9.25 | 3.16 |
| 34 | 4.92 | 6.11 | 7.83 | 6.84 | 8.29 | 3.68 |
| 35 | 6.22 | 5.99 | 8.34 | 4.71 | 12.17 | 5.09 |
| 36 | 4.73 | 6.02 | 11.18 | 7.43 | 4.09 | 3.13 |
| 37 | 4.11 | 4.84 | 7.95 | 5.84 | 11.45 | 2.32 |
| 38 | 4.35 | 8.35 | 9.63 | 7.11 | 2.72 | 5.72 |
| 39 | 2.65 | 4.37 | 10.22 | 6.16 | 4.56 | 4.24 |
| 40 | 2.93 | 4.71 | 8.56 | 3.68 | 2.08 | 3.71 |
| 41 | 4.70 | 3.24 | 7.34 | 3.97 | 5.74 | 5.80 |
| 42 | 4.67 | 2.82 | 5.75 | 9.98 | 3.96 | 2.35 |
| 43 | 2.18 | 6.85 | 4.95 | 6.21 | 4.72 | 6.50 |
| 44 | 2.50 | 5.96 | 6.84 | 7.62 | 2.42 | 2.41 |
| 45 | 6.21 | 6.49 | 7.66 | 7.18 | 4.31 | 4.07 |
| 46 | 7.28 | 5.43 | 6.15 | 7.64 | 8.94 | 6.29 |
| 47 | 5.39 | 6.69 | 4.22 | 4.06 | 4.45 | 4.34 |
| 48 | 6.02 | 6.76 | 8.24 | 4.72 | 4.81 | 4.32 |
| 49 | 7.03 | 4.61 | 4.32 | 4.02 | 2.11 | 5.50 |
| 50 | 9.02 | 3.08 | 6.30 | 8.29 | 7.33 | 6.60 |

a. “114-159” and “157-159” represented the salt bridges Asp114-Lys159 and Asp157-Lys159.

b. The value was the distance of salt bridges (Å) and the background was was shown as colored gradient from red to white to blue according to the value from 2.08 to 12.25.

**Table S3** Some thermostable endoglucanases from various microorganisms

| Microorganisms | Name of enzyme | Thermostability at temperature |
| --- | --- | --- |
| Bacillus | BsCel5A | 70% activity at 75 °C for 30 min or even less |
| Geobacillus sp. 70PC53 | GsCelA | 70% activity at 75 °C after 4 h |
| Geobacillus sp. HTA426 | CMCase | Stable at 50–70 °C for 5 h |
| Bacillus sp. SR22 | Bc22Cel | 30% of the activity retained at 80 °C at high salt molarity (1.5 M NaCl) |
| Humicola grisea | Egl2 | 80% residual activity for 10 min at 75 °C |
| Humicola grisea | egl3 | 75% residual activity for10 min at 80 °C |
| Humicola grisea | egl4 | 75% residual activity for10 min at 80 °C |
| Humicola grisea var thermoidea | Egl and cbh1 | Stable for 10 min at 60 °C and 55 °C respectively |


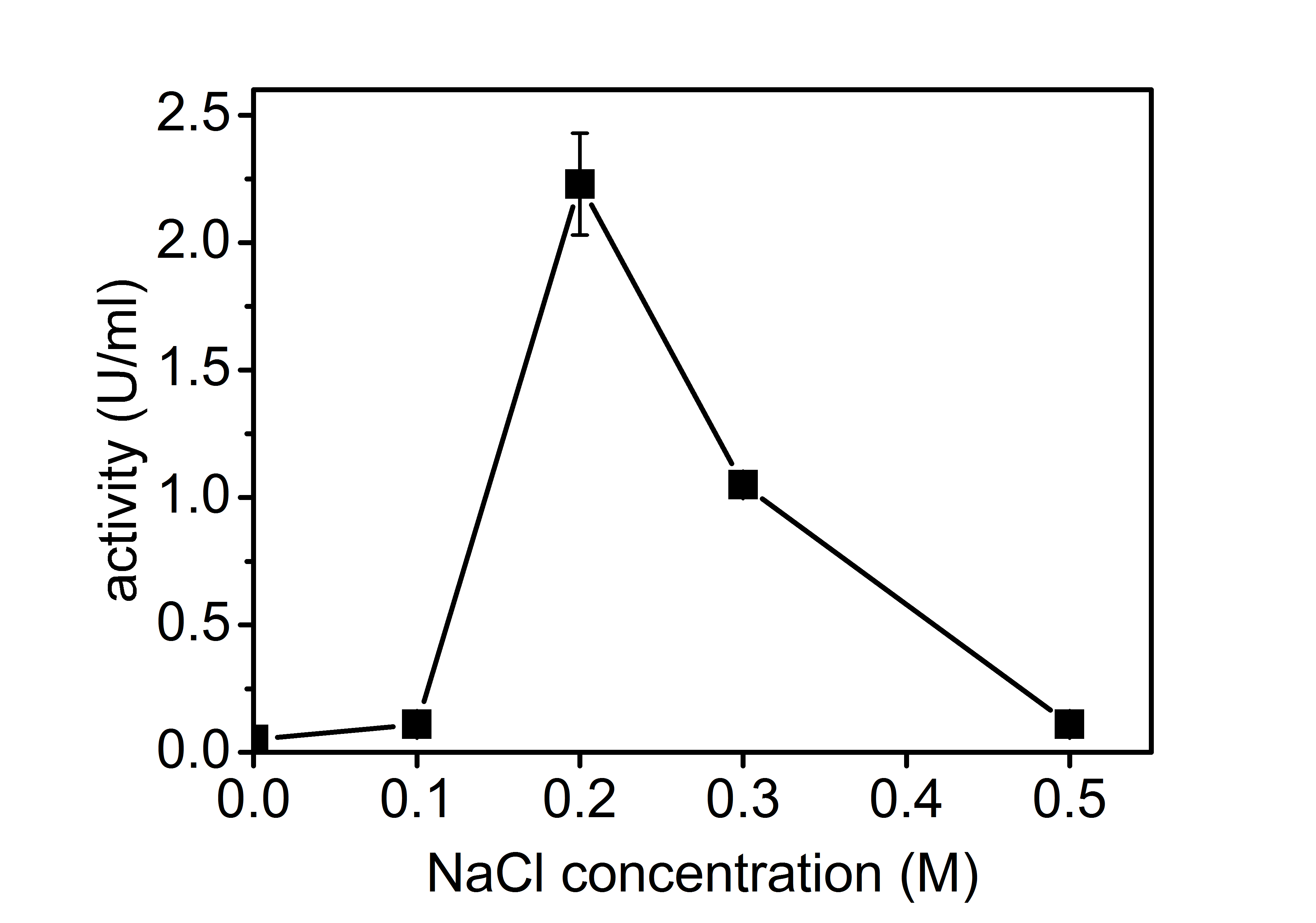


**Figure S1** NaCl gradient elution to the supernatant of original strain

Each eluent with different NaCl concentration was diluted to the same volume.


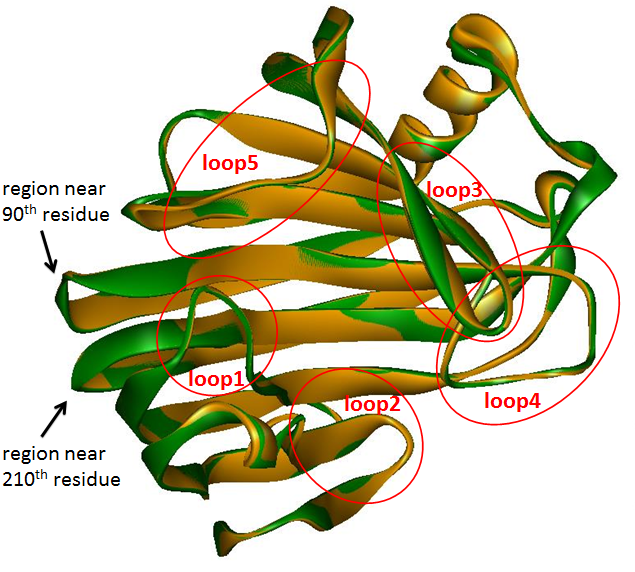


**Fig. S2** The positions of the regions near the 90^th^ and 210^th^ residues


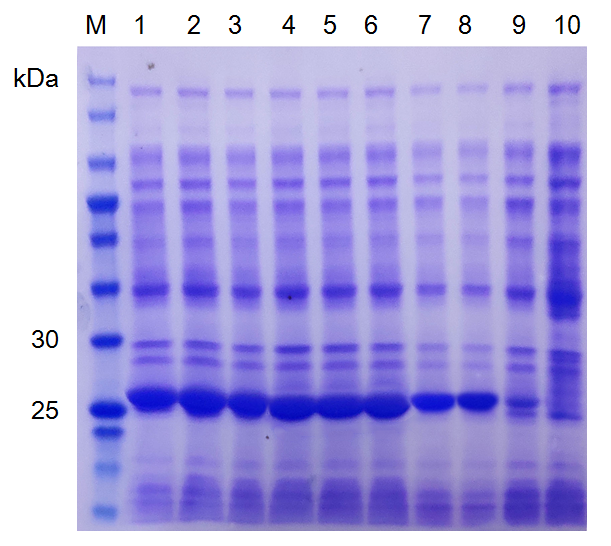


**Figure S3** The expression of *An*EGL and its mutants in *E. coli*

M: protein marker, 1: original *E. coli* Rosetta(DE3) induced by IPTG, 2-9：positive transformants with mutant 1-8 induced by IPTG, 10: original *E. coli* Rosetta(DE3) without induction


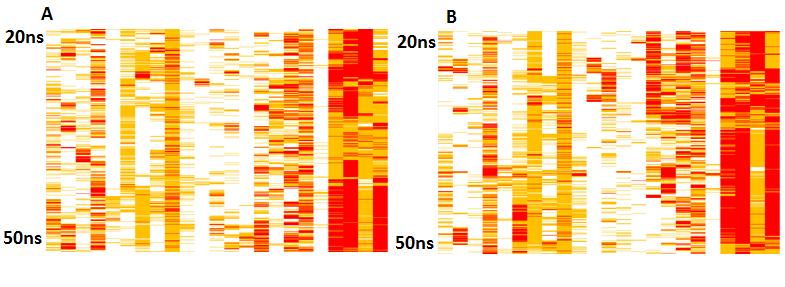


**Fig. S4** Changes of salt bridges within 20-50 ns simulation

(A) In the absence of NaCl (B) In the presence of 4 M NaCl.

Salt bridges with atomic distance less than 4 Å were defined as strong effect and shown in red, those with atomic distance between 4 and 6 Å were defined as secondary strong effect and shown in orange, and those with atomic distance greater than 8 Å were defined as weak effect and shown in white. Each column represented the strength change of each salt bridge within 20-50 ns simulation. The four columns on the right side of (A) and (B) were the strength changes of Asp95-Na^+^, Asp99-Na^+^, Glu116-Na^+^ and Glu204-Na^+^, respectively, and other columns were the strength changes of salt bridges between residues
